# Lateral forces determine dimensional accuracy of the narrow-kerf sawing of wood

**DOI:** 10.1038/s41598-021-04129-3

**Published:** 2022-01-07

**Authors:** Kazimierz A. Orlowski, Daniel Chuchala, Marcin Szczepanski, Wojciech Migda, Wiktoria Wojnicz, Jakub Sandak

**Affiliations:** 1 Institute of Manufacturing and Materials Technology, Gdansk University of Technology, Faculty of Mechanical Engineering and Ship Technology, Narutowicza 11/12, Gdansk, Poland; 2 EkoTech Center, Gdańsk University of Technology, Narutowicza 11/12, 80-233 Gdańsk, Poland; 3 Gdansk University of Technology, Faculty of Civil and Environmental Engineering, 80-233 Gdansk, Poland; 4 Institute of Mechanics and Machine Design, Gdansk University of Technology, Faculty of Mechanical Engineering and Ship Technology, Narutowicza 11/12, Gdansk, Poland; 5 InnoRenew CoE, Livade 6, 6310 Isola, Slovenia; 6 Andrej Marušič Institute, University of Primorska, Koper, Slovenia

**Keywords:** Mechanical engineering, Civil engineering

## Abstract

The shrinking global forest area limits the supply of industrially usable raw resources. This, in combination with the ever-increasing consumption of timber due to population growth can lead to the lack of a positive balance between the annual volumetric growth and consumption of wood. An important innovation toward increasing environmental and economic sustainability of timber production is to reduce the volume of wood residues by minimizing the sawing kerf. It results in higher material yield but may impact the dimensional accuracy of derived products. Therefore, the cutting tool geometry as well as the sawing process as a whole must be carefully optimized to assure optimal use of resources. The goal of this study is to better understand the causes of machining errors that occur when sawing wood with saws of varying thickness of kerf, with a special focus on re-sawing thin lamellae performed on the gang saw. Numerical simulations were tested against experimental results, considering influence of diverse components of cutting forces, in addition to the initial and operating stiffness coefficients of the saw blade. It has been demonstrated that asymmetric loads from the cutting process for the scraper saw blade can cause sawing inaccuracies. The simulation methodology developed in this research can be straightforwardly extended towards determination of optimal geometry of other cutting tools, particularly with the reduced sawing kerf. This may lead to more sustainable use of natural resources as well as an increase in economic gain for the wood processing industries.

## Introduction

The sawing of logs into timber and the following re-sawing of square-sawn timbers into lamellae are the most common basic woodworking operations performed in primary and secondary wood processing plants. The cost structure within a wood processing company is one of the most important factors determining its revenue. The material component associated with the sawn raw material has, in a typical European sawmill, the largest share in the cost structure reaching up to 70%^1^. Steele et al.^2^ argue that material costs can amount to even more, exceeding 75% in extreme cases. At the same time, the global forest area is shrinking, limiting the supply of industrially usable raw resources. This, in combination with the ever-increasing consumption of wood because of a growing population, can lead in the coming years to the lack of a positive balance between the annual volumetric growth and consumption of wood^3^. Projected and actual scarcity of the raw material (logs) availability, increasing competition on the international market, combined with significant and ever-growing production costs compel wood processing plants to dramatically modernize their technologies^4,5^. An important innovation toward increasing environmental and economic sustainability of timber production is to reduce the volume of wood residues by minimizing the sawing kerf. It results in higher material yield but may have an impact on the dimensional accuracy of derived products. Therefore, the cutting tool geometry as well as sawing process conditions must be carefully optimized to assure optimal use of resources.

The process of cutting wood on band sawing machines was experimentally studied^6,7^, while the accuracy of the sawing of timber under industrial conditions was evaluated^8^. Steele et al.^2^ analysed 266 reports for 6 types of band sawing machines and the accuracy of products obtained from them. Recommendations have been developed for the selection of sawing machines for cutting selected hardwood species, based on the measured values of the total sawing variation^9^. Barcik^6^, Lehmann and Hutton^10^, Tanaka et al.^11^ and Wong and Schajer^12^ reported results of their studies on the transverse movement of band saw blades during the cutting of wood. Albrecht and Möhring^13^ have demonstrated that adaptive control of the cutting of metals using band sawing machines improves the energy efficiency of the process, as well as cutting accuracy and machining productivity. The same research direction was also adopted for the material-efficient processing of wood^14^.

Ulsoy et al.^15^ analysed the dynamics and stability of band sawing machines. Similar issues were also addressed by Yang and Mote^16^, Lehmann and Hutton^10,17^, Lister and Schajer^18^ and Okai et al.^19^. The latter work explains the phenomenon of self-excited vibrations, called “washboarding”. It directly linked resonance frequencies of saw blades with the generated surface topography. Conversely, Orlowski and Wasielewski^20^ postulated that unwanted surface washboarding effect after cutting on a frame sawing machine is a result of the regeneration of vibrations or trace machining.

Other direction of wood cutting process studies is focused on circular saw blades. These can be fixed in clamp flanges or guided. Natural frequencies of saws and their dependent critical rotational speeds were determined in^21–24^. The impact of the design of the circular saw blade on its dynamic characteristics was described by Cheng and others^25^. The benefits of using circular saw blades running in guides at supercritical rotational speeds have been presented in several works^26–29^. In addition to experimental studies, numerical studies were also conducted, such as the computational model proposed by Mohammadpanah and Hutton^26^ as well as the multi-point pressure tensioning process of the circular saw blade^30^.

Issues in the production of top layers of adhesive-bonded floors on band sawing and frame sawing machines using saws with small values of the overall saw set (theoretical kerf) were analysed in the work of Orlowski and Walichnowski^31^. Kujawińska et al.^32^ and Zywicki et al.^33^ proposed a model for estimating raw material waste during the production of the middle layers of the 3-layer floorboard. Similarly, the optimization of raw material allowances in the production of thin lamellae from selected European and exotic wood species, by implementing the “wet technology” on band sawing machines was studied by Kujawińska et al.^34^. The methodology for determining optimal allowances for processing, and application of the methodology in the process of manufacturing oak wood products for the top layer of adhesive-bonded floorboards are described in the article^35^.

**Figure 1:**
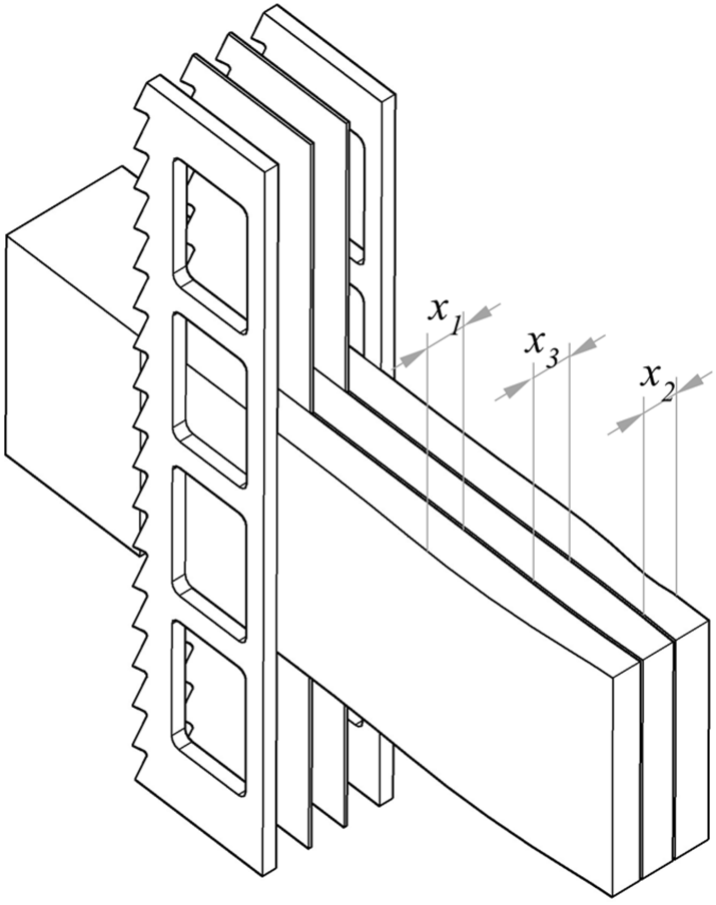
The set of saw blades in the gang with the obtained lamellae (drawing designed in AutoCad 2020 https://www.autodesk.com/products/autocad).

The standard deviations within-lamella, between lamella*,* and total standard deviations, are accepted as reliable quantifiers of the accuracy of the sawing process^9,36^. It was evidenced by authors^37^ that variations in thickness for the external lamellae *x*_1_ and *x*_2_ were consistently higher than for the middle lamellae *x*_3_ (Fig. 1), and increased along the working shift. The accuracy assessment was carried out using the discrete method^37^, though solutions based on artificial intelligence algorithms could also be used for monitoring the cutting process^38^. The asymmetric action of the cutting forces on the main cutting edges and the action of the thrust forces on the minor cutting edges of the scraper saw blades are thought to be the main cause of this dimensional inaccuracy of the external lamellae. This phenomenon occurred despite the considerably larger thicknesses of lateral saw blades compared to the middle saws in the gang.

The goal of this study is to better understand the causes of machining errors that occur when sawing wood with saws of varying thickness of kerf. It could lead to the development of a universal protocol for the determination of optimal saw geometry resulting in a minimal amount of generated residues (saw dust) while assuring tolerable dimensional accuracy. The case study presented is a follow-up of the long-term experimental campaign conducted by authors in the past. It includes studies on the effectiveness of frictional narrow-saw blade fastening in the gang^39^, methods of determining static saw blade stiffness^40^, washboarding phenomenon and dynamic behaviour of narrow-kerf saw blades while sawing soft and hard woods^20^, economic analyses of lamellae production methods^31^, quality of re-sawing process^37,41^, application of fracture mechanics for determination of energetic effects while wood sawing^42,43^, and dynamics of the main driving system of the sash gang sawing machine^44^. This work focuses on an analysis of the re-sawing processes of thin lamellae performed on the gang saw when implementing narrow-kerf saws. Numerical simulations were tested against experimental results, considering influence of diverse components of cutting forces, in addition to the initial and operating stiffness coefficients of the saw blade.

## Theoretical background

### Stiffness of the saw blade

**Figure 2:**
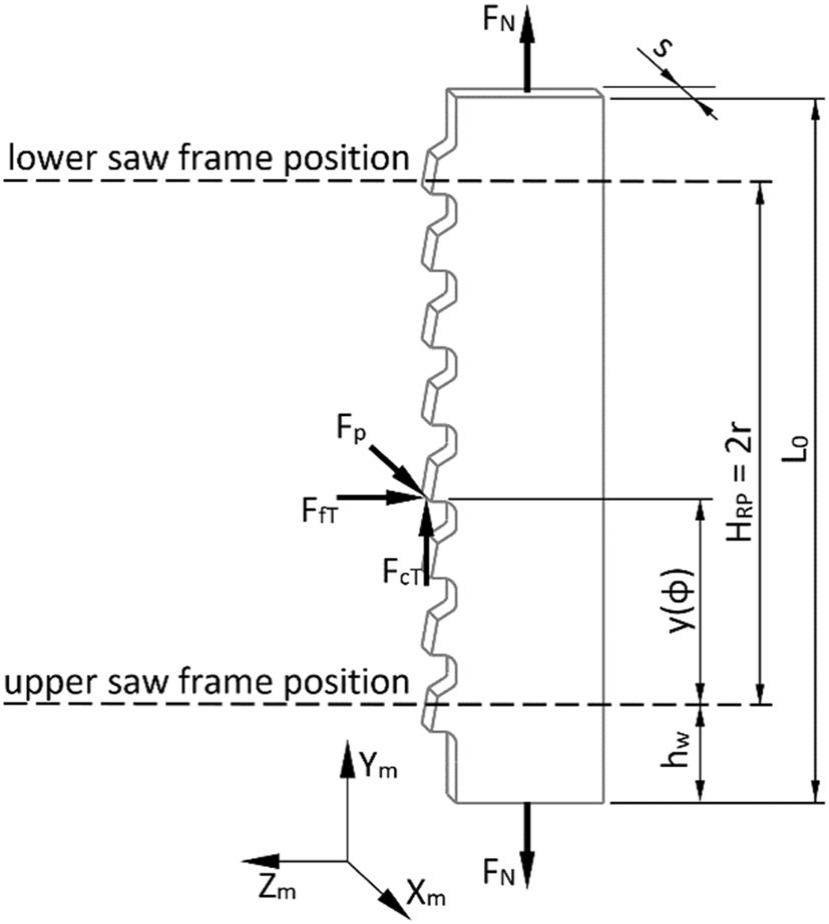
Loads on the saw during operation; where: *F*_*N*_ is the tensioning force (the force stretching the saw blade in the longitudinal direction—Y_m_ axis of the sawing machine’s coordinate system); *F*_*cT*_ is the total cutting force; *F*_*fT*_ is the total feed force; *F*_*pT*_ is the total thrust force to load the saw; *L*_0_ is the free saw blade length; *h*_*w*_ is the feed roller level; *H*_*RP*_ is the saw frame stroke; *r* is the crank radius in the driving system; *s* is the saw blade thickness; *y(φ)* is the position of the point of application of force as a function of the angle of rotation *φ* of the crank in the main drive of the sawing machine.

The static stiffness of the saw determined in the machine–chuck–workpiece–tool system, combined with the resistance to wandering in the workpiece, are commonly considered as the basic indicators determining the ability of a saw to properly cut the material^10,17,45^. Therefore, the saw in the given configuration represents the weakest element of the system. Several attempts were made to predict the behaviour of the tool during the machining^5,27^. Most of these revealed that the geometrical accuracy of the products being manufactured depends on the transverse displacements of saw blades. Figure 2 shows an arrangement of external forces loading the saw blade, which change their positions depending on the placement of the frame sawing machine sash, when it is driven by a dynamically balanced main drive^44^.

The initial static stiffness of the saw blade *k*_0_ is determined according to Eq. (1) as the ratio of the thrust force *F*_*p*_ applied in the middle of the free length of the saw blade to the displacement of the saw blade *q* towards the X_m_ axis (Fig. 2):

1 $$ k_{0} = \frac{{F_{p} }}{q} $$

The relationship (1) is non-linear as the stiffness of the saw blade *k*_0_ is a nonlinear property. Therefore, the stiffness itself should be assessed as a local stiffness by using the secant method. In that case the ratio of the gain in the thrust force is directly related to the gain in the displacement of the saw blade^46^. The stiffness of the saw blade is most often analysed by the superposition method, which accounts for the strain of the saw blade resulting from bending (normal strains due to bending) and torsion (shear strains due to torsion)^40,47–51^. Several analytical methods (the beam and plate models) and/or numerical methods (the finite element method or the rigid finite element method) are frequently used to determine the static stiffness values. However, each of those methods can only be used within the specified range of blade width^40^. The saw blade width can vary between 20 mm in the case of mini saw blades, up to > 250 mm for high throughput band sawing machines for processing logs.

### Factors influencing the saw blade stiffness

An effect of the feed force *F*_*f*_ should be taken into consideration when analysing transverse displacements of the saw. *F*_*f*_ affects the stability of the saw blade and reduces the initial static stiffness coefficient of the saw blade *k*_0_ to the value of the operating stiffness coefficient *k*_0*w*_^24,49,51^. The total loss of saw blade stability occurs when the feed force *F*_*f*_ reaches the critical value (*F*_*f*_*crit*_) operating in the plane of the greatest stiffness of the saw blade^50–52^. The critical force *F*_*f*_*crit*_ corresponds to the force at which the saw blade gets infinitely bent as a result of a small value thrust force *F*_*p*_^11,50,51^. In addition, the stiffness of the saw blade can decrease as a result of an increase in the saw blade temperature^53^. Pahlizch and Puttkammer^50^, Prokofiev^51^ and Csanady and Magoss^47^ proposed the analytical determination of the critical feed-per-tooth force *F*_*f*_*crit*_ for saws with saw blades wider than 100 mm.

Knowing the value of the critical force *F*_*f*_*crit*_, the working stiffness coefficient can be determined from the dependencies proposed by Timoshenko and Gere^52^ for the general case, or by Prokofiev^51^ specifically for a wideband sawing machine. Finally, the working stiffness can also be derived from Stakhiev’s equation^24^.

### Estimating values of cutting forces

The cutting forces *F*_*c*_ can be estimated using the empirical classic model which is based on wood specific cutting resistance^43,54–56^. As an alternative, a modern approach considering the fracture toughness and shear yield stress in the cutting zone can be used for the determination of cutting force values^43^. Although models based on the fracture mechanics theorem allows for predicting energy effects in a highly precise way, the classical empirical method is still widely used in solving practical problems. For this reason, it was adopted for the numerical simulations developed within this research, in which the cutting force *F*_*c*_ was described as in Eq. (2):

2 $$ F_{c} = k_{c} \cdot A_{Dav} = k_{c} \cdot S_{t} \cdot h_{av} $$

where: *k*_*c*_ is the coefficient describing the specific cutting resistance [N⋅mm^−2^], *F*_*c*_ is the cutting force [N]; *A*_*Dav*_ is the mean cross-section area of the uncut chip [mm^2^]; *S*_*t*_ is the total kerf [mm]; *h*_*av*_ is the mean uncut chip thickness [mm].

This model shall consider experimentally determined correction coefficients identified to address changes in the factors affecting the cutting process in relation to the basic conditions adopted in^56^, as summarized in Eq. (3):

3 $$ k_{c} = k_{\phi } \cdot c_{ws} \cdot c_{MC} \cdot c_{vc} \cdot c_{\delta } \cdot c_{d} \cdot c_{wT} \cdot c_{h} \cdot c_{\mu } \cdot c_{CE} \cdot c_{p} $$

where: *k*_*φ*_ is the basic specific cutting resistance for Scots pine wood [N·mm^-2^], *c*_*ws*_ is the coefficient taking into account the wood species (i.e. *c*_*ws*_ = 1 for Scots pine wood defined as the reference species); *c*_*MC*_ is the coefficient correcting effect of the wood moisture content; *c*_*vc*_ is the coefficient taking into account the value of the cutting speed; *c*_*δ*_ is the coefficient taking into account the cutting angle defined as the sum of the clearance angle *α*_*f*_ and the blade angle *β*_*f*_; *c*_*d*_ is the coefficient considering the cutting edge wear; *c*_*wT*_ is the coefficient adjusting effect of the temperature of the wood; *c*_*h*_ is the coefficient taking into account the value of the uncut chip thickness; *c*_*µ*_ is the coefficient taking into account the friction between the wood being cut and the saw blade; *c*_*CE*_ is the coefficient taking into account the shape and dimensions of the cutting edge; *c*_*p*_ is the factor taking into account the pressure exerted on the workpiece in front of the blade (commonly applied in the production of veneer).

The value of *k*_*φ*_ depends on the position of the cutting edge in relation to the direction of the wood grains^56^. These values take into account the basic cutting directions, along the grains (*k*_*II*_), tangentially to the grains (*k*_*#*_) and perpendicularly to the grains (*k*_*⊥*_), as well as intermediate cutting directions *(k*_*II#*_*, k*_*II⊥*_*, k*_*#⊥*_*, k*_*II#⊥*_). The values of all the correction factors are equal to 1 if the cutting process is carried out under basic reference conditions. In that case, the specific cutting resistance *k*_*c*_ corresponds to the basic specific cutting resistance *k*_*φ*_ (Eq. 4):

4 $$ k_{c} = k_{\phi } \cdot 1 = k_{\phi } $$

### Lateral loads of the saw blade during cutting

Saws with spring setting teeth, especially those with narrow kerfs, are not used for precise cutting as they do not have the symmetry of tooth geometry for the minor cutting edges. Thus, it is important to properly assess the cause of the build-up of the thrust force on saws with swaged teeth. Some of these tools have already been analysed, including circular saw blades^57^ and frame saw blades^51^. The causes of build-up of the resultant thrust force on the teeth of the saw with the swaged saw set are presented here in a structured manner, referring to the said works as well as the requirements given in the ISO standards (ISO 3002-1:1982^58^ and ISO 3002-2:1982^59^).

**Figure 3:**
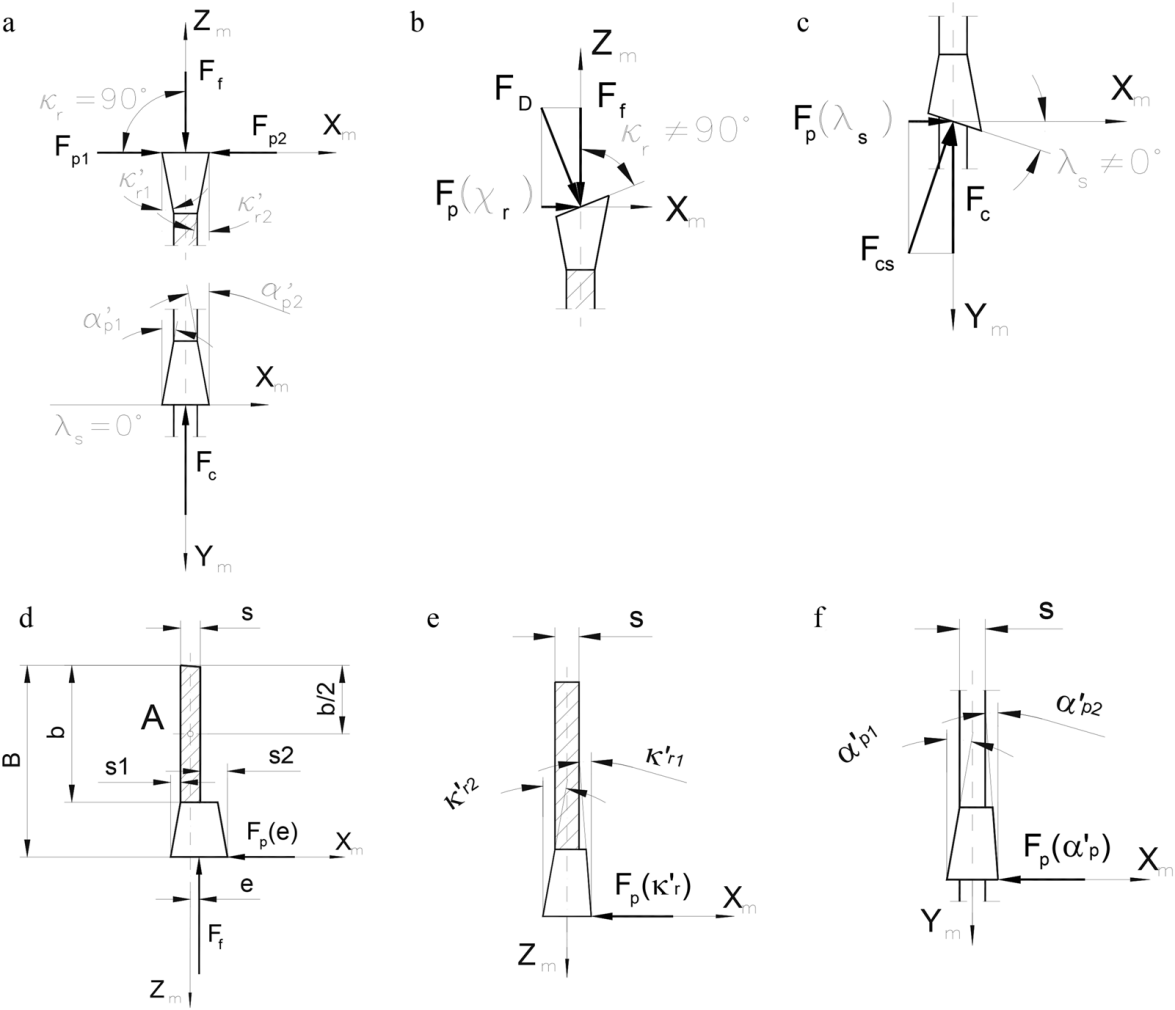
Transverse loads of the saw blade as a function of its geometry in the sawing machine’s coordinate system (X_m_, Y_m_, Z_m_): **(a)** typical blade with the swaged saw set; **(b)** effect of the cutting edge angle *κ*_*r*_; **(c)** effect of the angle of inclination of the main cutting edge *λ*_*s*_; **(d)** effect of the difference in the values of the side saw sets *s*_*2*_ > *s*_*1*_; **(e)** effect of the difference in the values of tool minor cutting edge angles *κ*_*r1*_*'* < *κ*_*r2*_*'*; **(f)** effect of the difference in the values of the rear angles of clearance *α*_*p1*_*’* > *α*_*p2*_*’*.

A typical tooth with the swaged saw set is schematically presented in Fig. 3a. The cutting edge angle and the inclination angle of the main cutting edge are *κ*_*r*_ = 90° and *λ*_*s*_ = 0°, respectively. The minor cutting edge angles and the rear flank angles on both sides of the blade are the same (*κ*_*r1*_*'* = *κ*_*r2*_*', α*_*p1*_*'* = *α*_*p2*_*'*). Both thrust forces *F*_*p1*_ and *F*_*p2*_ should be balanced in such a configuration of tooth geometry.

Saw blades with a cutting edge angle *κ*_*r*_* ≠ 90°* are additionally loaded with an extra thrust force *F*_*p*_(*κ*_*r*_). Its value depends on the feed force *F*_*f*_ (Fig. 3b) and the additional thrust force *F*_*p*_(*λ*_*s*_) caused by the cutting force *F*_*c*_ (Fig. 3c) when the main cutting edge is tilted at an angle *λ*_*s*_* ≠ 0°*.

Grinding errors in the form of large differences between the values of the side sets *s*_*1*_ and *s*_*2*_ have a significant impact on the resultant thrust force in addition to the assumed geometry of the saw blades. Such an impact is directly related to the presence of an additional thrust force *F*_*p*_(*e*), as shown in Fig. 3d.

Another common error occurring during the manufacture of saw blades is the lack of symmetry due to differences in the minor cutting edge angles, *κ*_*r1*_*' ≠ κ*_*r2*_*'* (Fig. 3e) and/or in the rear flank angles *α*_*p1*_*' ≠ α*_*p2*_*'* (Fig. 3f). Each of these errors results in the presence of an additional thrust force *F*_*p*_(*κ*_*r*_*’*). *F*_*p*_(*α*_*p*_*’*), leading to a variable thickness in the generated workpieces.

An additional cause of the asymmetrical forces in the saw working system can be the inaccurate positioning of blades within the saw frame. An additional thrust force will appear when the direction of the feed movement (or the feed force vector *F*_*f*_) and the Y_m_-Z_m_ plane of the setting system are divergent^60^.

It should be stressed, however, that besides the above-listed factors, the natural heterogeneity of the processed material is the driving factor resulting in the unbalance of the thrust forces *F*_*p1*_ and *F*_*p2*_. It is especially relevant in the case of processing biological origin materials, such as wood.

## Reference case study

The subsequent numerical simulations of the cutting process are an extension of the previously reported experiments where determination of the cutting power^42,43^, dimensional accuracy^37,41^ and saw blade stiffness^40^ were investigated. It corresponded to the thin lamellae re-sawing on the frame sawing machine with the gang configuration as presented in Fig. 1. The list of relevant case study settings is summarized below.

### The processed material

Analyses of the stiffness and stability of saw blades were carried out on the frame sawing machine for processing oak wood (*Quercus* L.). The thickness of re-sawed boards was *t*_*b*_ = 24 mm, with 200 mm width and 2000 mm board length. The moisture content of the wood during sawing was MC = 10%, resulting in the proceeding kiln drying process. Three thin lamellae of thickness *t*_*l*_ = 6 mm were obtained as a result of the sawing process. The expected tolerance of the thickness deviations was <  ± 0.2 mm. Such lamellae are used for manufacturing the top layers for multi-layer adhesive-bonded wooden floorboards.

### Sawing machine

Mamuth TR 97 frame sawing machine (Neva, CZ) equipped with a channel system for the simultaneous feeding of boards served as an experimental cutting platform. The basic parameters of the frame sawing machine are summarized in Table 1. The sawed lamellae thickness accuracy analysis was carried out for one of the channels leading the material to be sawn^37^. The studied channel consisted of two narrow-kerf saw blades (kerf width *S*_*t*_ = 1.4 mm) and two scraper saw blades (*S*_*t*___sc_ = 2.9 mm). The thickness of the scraper saw blades was s_sc_ = 2.4 mm, while that of the narrow-kerf saw blades was *s* = 0.9 mm. Teeth in both saw blade types were stellited and have the same tooth geometry within the set (Table 2). The sawing process was analysed for kinematic parameters used daily in the Łąccy-Kolczygłowy Sp. z o.o. sawmill in Barnowo (Pomerania, Poland). The results are presented in Table 2.

**Table 1:** The basic parameters of the frame sawing machine Mamuth.

Parameter	Value	Unit
Stokes per minute, *n*_*F*_	450	spm
Feed speed, *v*_*f*_	0.2–2.0	m·min^−1^
Power of the main motor, *P*_*EM*_	22	kW
Frame saw stoke length, *H*_*RP*_	250	mm
Cutting height, *H*_*P*_	250/320	mm
Cutting width, *W*_*P*_	220	mm
Width of kerf, *S*_*t*_	1.3–1.8	mm
Minimum lamellae thickness, *t*_*l*___*min*_	2.0	mm
Minimum board length, *L*_*b*___*min*_	250	mm

**Table 2:** The parameters of the sawing process used for the analysis.

Parameter	Value	Unit
**Sawing process**		
Cutting speed, *v*_*c*_	3.75	m·s^-1^
Feed speed, *v*_*f*_	0.3–0.5	m·min^-1^
Feed per tooth, *f*_*z*_	0.04	mm
Uncut chip thickness, *h*_*av*_	0.04	mm
Cutting height, *H*_*P*_	200	mm
**Cutting tool**		
Pitch of teeth, *P*	15	mm
Tool side rake, *γ*_*f*_	8	
Tool side flank, *α*_*f*_	11	
Tool cutting edge angle, *κ*_*r*_	90	
Cutting edge inclination angle, *λ*_*s*_	0	
Width of saw blade body, *b*	35	mm
Width of saw blade, *B*	40	mm
Number of teeth, *z*	31	–
Free length of the saw blade, *L*_*0*_	550	mm

### Cutting tools

All the saw blades were fixed together in the gang, using a friction force-based mechanism similar to the solution described in the report^39^. The procedure for fixing the saw blades included the pre-tensioning of the entire saw blade assembly by providing a force of *F*_*N*_ = 9450 N on each blade (Fig. 2). The value of the force was determined assuming the normal tensile stress *σ*_*N*_ in the narrow-kerf blade corresponding to 300 MPa, following Eq. 5:

5 $$ F_{N} = \sigma_{N} \cdot A_{b} $$

where: *A*_*b*_ is the cross-section of the body of the narrow-kerf saw blade calculated as a product of the thickness *s* and width *b*.

Bodies of the scraper saw blades were much thicker than those of the narrow-kerf saw blades installed in the centre of the set. Rectangular windows were cut out in scraper saw blades to assure a balanced value of normal tensile stress corresponding to that in the narrow-kerf saws (Fig. 1). The width of the window was *b*_*w*_ = 14 mm, and its length was *l*_*w*_ = 33 mm. Consequently, the stress in the scrapper saw blades within the smallest cross-section at the window zone was *σ*_*N*___sc_ = 187.5 MPa.

**Figure 4:**
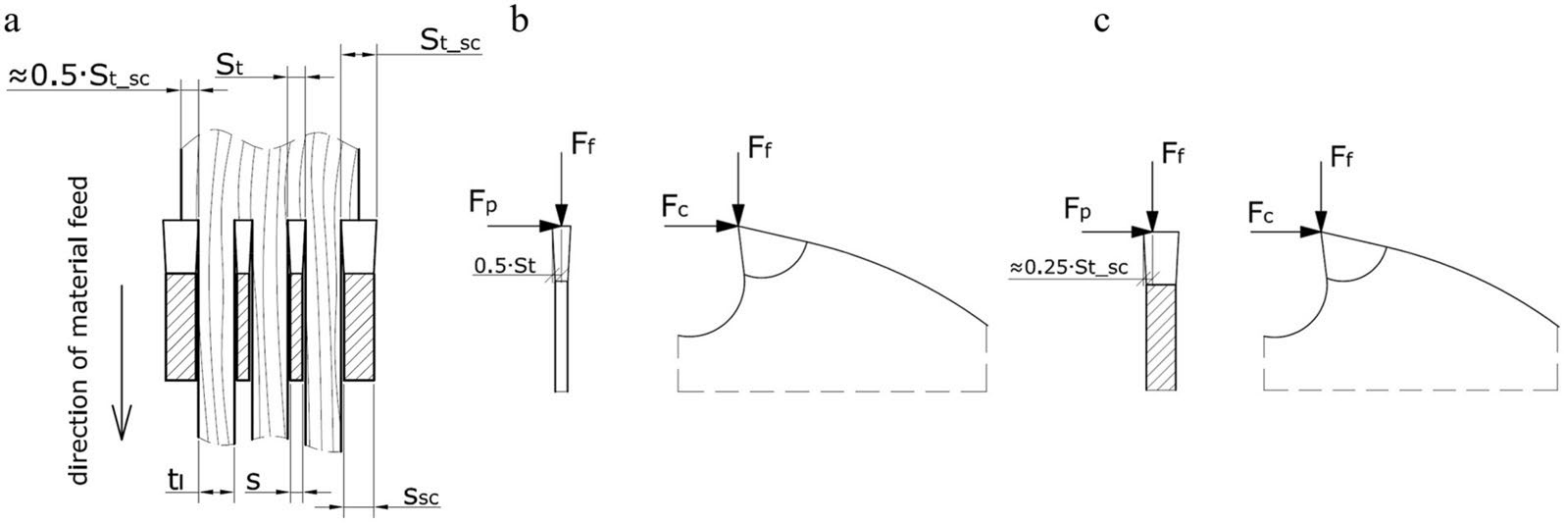
The arrangement of the saw blades in the gang together with the operating range of the saw blades depending on the position of the saw blade in the gang **(a)**. The place of application of concentrated forces loading the cutting blades: the narrow-kerf saw blade **(b)** and the scraper saw blade **(c)**.

Given that both types of saw blades (within the same channel) worked under different cutting conditions during the sawing process, it was assumed that (Fig. 4a):

• The two narrow-kerf saw blades located in the middle of the set carried out the cutting process with the entire length of the main cutting edge (*S*_*t*_ = 1.4 mm), and the load forces were concentrated halfway along the cutting edge of each saw blade (Fig. 4b).

• The two scrapper saw blades located on both sides of the set carried out the cutting process with only half of the length of their main cutting edges (*S*_*t*___*sc*_/2 = 1.45 mm), and the load forces were concentrated in one-quarter of the length of the cutting edge of each saw blade (Fig. 4c).

• Besides the main cutting edges, two minor cutting edges were involved in cutting with narrow-kerf saw blades, while only one minor cutting edge in the case of scrapper saw blades.

## Setting the sawing process simulation

### Determination of cutting forces when processing experimental samples

The value of the basic specific cutting resistance for the analysed cutting direction (perpendicular to fibres) corresponds to *k*_*φ*_ = *k*_*⊥*_ = 53 MPa^56^. The values of other correction coefficients, as used in Eq. (3) for calculating the value of the specific cutting resistance according to the classic method of Orlicz^56^ are summarized in Table 3.

**Table 3:** Correction coefficients for the analysed cutting process conditions.

Correction coefficient	Reference basic conditions		Analysed conditions	
	Basic data	Value	Analysed data	Value
*c*_ws_	Pine wood (*Pinus sylvestris* L.)	1	Oak wood (*Quercus* L.)	1.5
*c*_MC_	Dry wood MC = 10/15%	1	Dry wood MC = 10/15%	1
*c*_vc_	Up to 10 m⋅s^-1^	1	3.75 m⋅s^-1^	1
*c*_δ_	60°	1	82°	2
*c*_d_	Sharp blade ρ_o_ = 4/10 μm	1	Sharp blade ρ_o_ = 4/10 μm	1
*c*_wT_	20 °C	1	20 °C	1
*c*_h_	h = 0.15 mm	1	h = 0.04 mm	1.7
*c*_µ_	Frame saw *H*_*P*_ = 120–160 mm	1	Frame saw *H*_*P*_ = 180–220 mm	1.05
*c*_CE_	Single cutting edge/frame saw	1	Frame saw	1

Values of the cutting force *F*_*c*_ computed using the above model are summarized in Table 4. These are relevant for the case study configuration and were directly used in the following simulations. Equivalent value of force F*_c_ determined during the real cutting test at a similar machining configuration is presented in Table 4 for comparison. It corresponds to the sawing process of oak wood at a feed speed of 0.5 m min^−1^^61,62^. It is evident that experimental and modelled values of cutting forces are comparable. However, it ought to be emphasised that energetic effects determined with the classic method are often over-estimated in comparison to experimental results or other models utilizing fracture mechanics theorem^43^.

**Table 4:** Total cutting forces *F*_*c*_ computed for the analysed cutting process conditions and corresponding F*_c_ determined experimentally.

Feed speed *v*_*f*_ (m·min^-1^)	Cutting force *F*_*c*_ (N)	Cutting force F*_c_ (N) Measured cutting force F_c_ (N)
0.3	212	–
0.4	250	–
0.5	287	261

### Determination of feed and thrust cutting forces when processing experimental samples

Specific values of the analysed feed forces *F*_*f*_ were computed in relation to the mean cutting force *F*_*c*_*av*_ determined for the mean feed rate simulated in this research (*v*_*f*_ = 0.4 m·min^−1^). It was found in the author’s own studies that the feed forces *F*_*f*_ can reach values equal to the cutting forces *F*_*c*_ or even higher when processing wood on the frame sawing machine. Therefore, the following values of the feed forces *F*_*f*_ were adopted for the numerical analysis: 0.5·*F*_*c*_*av*_ = 125 N, 1.0·*F*_*c*_*av*_ = 250 N and 1.5·*F*_*c*_*av*_ = 375 N.

Similarly, the set of the thrust forces *F*_*p*_ used for the analysis included 10 N, 50 N, 100 N, 150 N, 250 N and 375 N. It enabled numerical analysis to simulate diverse thrust force *F*_*p*_ effects covering a broad range of corresponding cutting force *F*_*c*_ values.

### Numerical modelling of saw blades

The numerical analysis of the static stiffness of the saw blades was carried out using the rigid finite element method RFEM software from Dlubal Software GmbH (Tiefenbach, Germany). It performs numerical analysis based on the finite element method, utilizing the iterative Picard algorithm^63^. This solution can solve non-linear problems and is characterized by improved numerical stability as compared to the Newton-Rapson method^64^.

**Figure 5:**
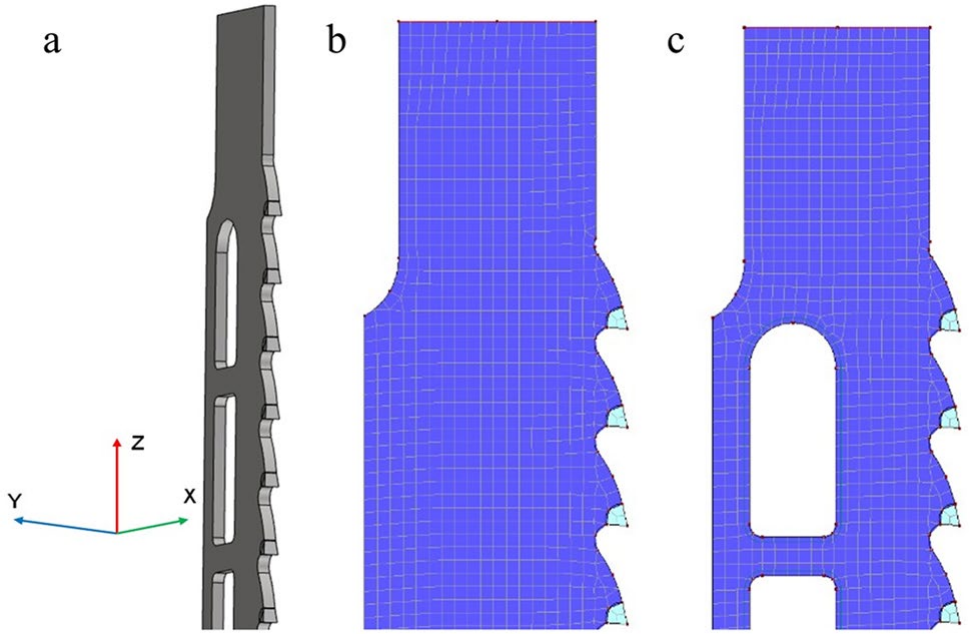
The 3D model with the global coordinate system **(a)** and finite element mesh of the analysed saw blades corresponding to the narrow-kerf blade **(b)** and the scraper saw blade **(c)** (drawing designed in AutoCad 2020 https://www.autodesk.com/products/autocad and Dlubal https://www.dlubal.com).

Separate numerical models were developed for the analysis of the narrow-kerf saw blade (Fig. 4b) and the scraper saw blade (Fig. 4c). Mechanical properties were modelled using isotropic models with material properties summarized in Table 5 according to^65,66^. Four-node (quadrangle) and three-node (triangle) finite shell elements of 6 degrees of freedom (DoF) were used to model the body of each saw blade, based on the Mindlin/Reissner theory^67^. The thickness of the elements was 0.9 mm and 2.4 mm for the narrow-kerf and for the scraper saw blade, respectively. This resulted in a model of the narrow-kerf saw blade consisting of 5218 2D elements combined with 5493 nodes and a model of the scraper saw blade consisting of 3851 2D elements combined with 4393 nodes (Fig. 5).

**Table 5:** Basic material properties of the analysed saw blades.

Element	Material	Density (g/cm^3^)	MoE (GPa)	Poisson ratio	Material model
Body of saw blade	Steel 1.2003	8.03	210	0.30	Isotropic, linear elastic 2D
Tooth	Stellite alloy	8.69	230	0.21	Isotropic, linear elastic 2D

**Figure 6:**
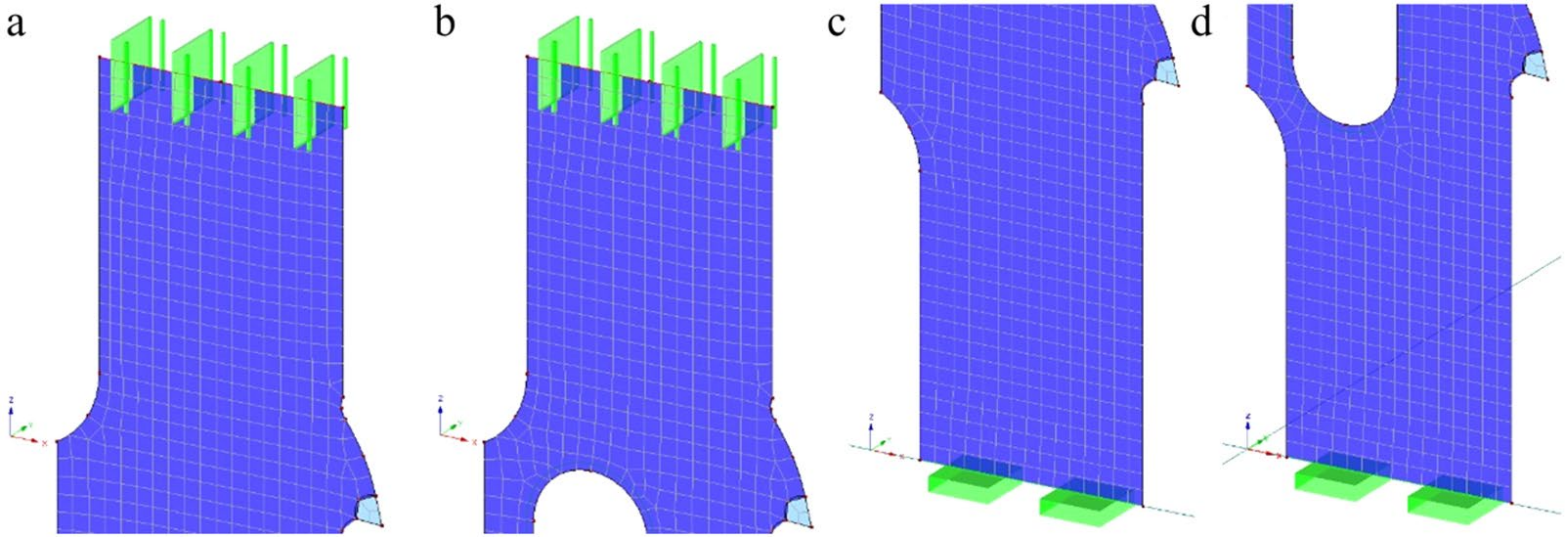
Boundary conditions for the simulated saw blades: top support for the narrow-kerf **(a)** and scraper saw blade **(b)**, the bottom support for the narrow-kerf **(c)** and scraper saw blade **(d)** (drawing designed in Dlubal https://www.dlubal.com).

All teeth were modelled in both saw variants in the same way as the bodies. Similar shell elements were used, differing only by an element thickness of 1.4 mm or 2.9 mm, respectively. Simulated saw blades were fixed with linear support along their edges on both ends (Fig. 6a,b) to simulate the original attachment to the saw frame. The lower end was fully immobilized to prevent any translation or rotation (Fig. 6c,d). Different support was applied to the upper end, in which displacements in the X and Y directions were blocked. The release in the global direction Z was applied to allow the introduction of the *F*_*N*_ force responsible for preloading the saw blades in this direction. The finite element method’s mesh for both saw blades was automatically generated by the software, using the following boundary meshing conditions: the target length of the elements = 2 mm, the maximum diagonal ratio = 1.8, and the maximum angle of tilt of a single quadrilateral element from the plane = 0.5°. The automatically generated mesh was adjusted to the geometry of the model, which greatly reduced the duration of computation.

Both saw blade models were preloaded by applying the force *F*_*N*_ = 9450 N assigned to the upper edge of the saw blade in the longitudinal direction (Z). A stiff 1D element was implemented to achieve an even distribution of the load over the entire width of the free edge of each blade.

### Loading configurations of modelled saw blades

Numerical analyses were carried out for each saw blade (Fig. 2) by implementing 78 variants of the loading configuration and corresponding cutting forces (*F*_*c*_*, F*_*f*_*, F*_*p*_) as summarized in Table 6. Loads from individual forces were concentrated at one point on the cutting edge of the medial tooth of the saw blade. This corresponded to the middle of the cutting edge in the case of the narrow-kerf saw blade (Fig. 4b). Alternatively, the point of application of the forces was located at 1/4th of the cutting edge’s width when modelling the scraper saw blade (Fig. 4c).

**Table 6:** Cutting forces *F*_*c*_ for diverse variants of feed *F*_*f*_ and trust *F*_*p*_ forces used for the numerical analysis of saw blades.

Feed force *F*_*f*_ (N)	Thrust force *F*_*p*_ (N)					
	10	50	100	150	250	375
0	0	0	0	0	0	0
125	0	0	0	0	0	0
250	0	0	0	0	0	0
375	0	0	0	0	0	0
125	212	212	212	212	212	212
250	212	212	212	212	212	212
375	212	212	212	212	212	212
125	250	250	250	250	250	250
250	250	250	250	250	250	250
375	250	250	250	250	250	250
125	287	287	287	287	287	287
250	287	287	287	287	287	287
375	287	287	287	287	287	287

## Results and analysis of the sawing process simulation

### Stiffness and stability of the saw blades

**Figure 7:**
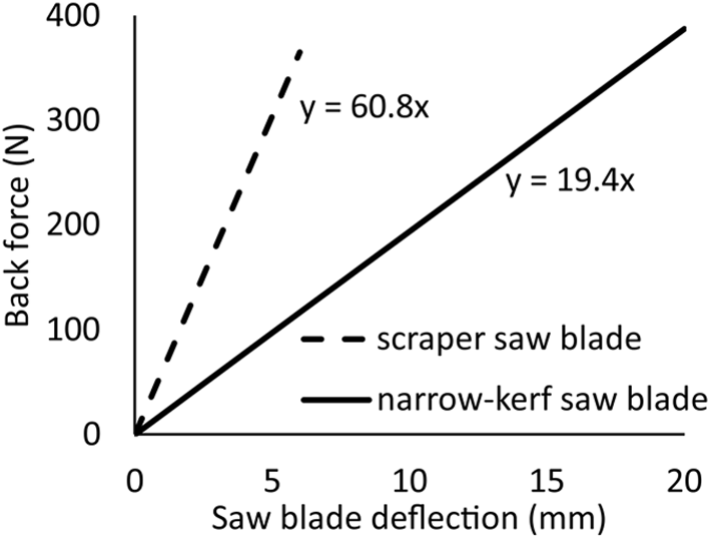
The elastic characteristics of the narrow-kerf and scraper saw blades. Note: each saw blade was tensioned with the force *F*_*N*_ = 9450 N.

The static stiffness values of the narrow-kerf and the scrapper saw blades were determined for loading the saw with the thrust force *F*_*p*_ in the middle of the free length of the saw blade. Additionally, the blade was subjected to the normal tensile force *F*_*N*_ = 9450 N. The characteristics of the elasticity of the saw blades linking the thrust force *F*_*p*_ to the strain *q* produced by this force in the direction of its action is shown in Fig. 7^46^.

Linear relationships for the load in the analysed range of variability of the thrust force *F*_*p*_ were obtained in both models. Therefore, slope factors in the trend line equations can be considered as coefficients of the initial static stiffness *k*_0_. It corresponded to *k*_0_ = 19.4 N⋅mm^−1^ and *k*_0_ = 60.8 N⋅mm^−1^ for the narrow-kerf and the scrapper saw blades, respectively. It revealed that the static stiffness of the scraper saw was more than three times higher than that of the narrow-kerf saw blade.

**Figure 8:**
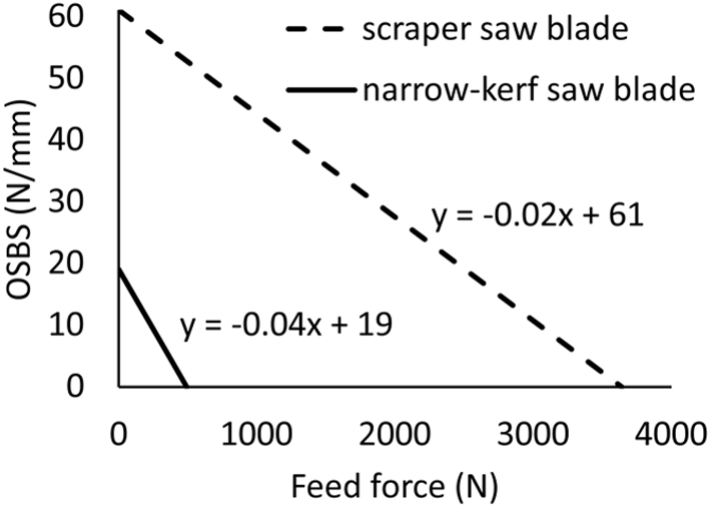
The operational saw blade stiffness *OSBS* of the narrow-kerf and the scraper saw blades expressed as a function of the feed force.

Figure 8 illustrates the operational saw blade stiffnesses *OSBS* for both the narrow-kerf and scraper saw blades. *OSBS* is calculated as an apparent result of the feed force *F*_*f*_. action. This force has a major impact on the stability of the saw blade and reduces the initial static stiffness coefficient *k*_0_ to the value of the operating stiffness coefficient *k*_*0w*_. The characteristics shown in Fig. 8 can be used to determine the critical feed forces at which the *k*_*0w*_ drops to zero. The critical feed force is equal to *F*_*f*_*crit*_ = 493 N in the case of the narrow-kerf saw blade. However, the critical force is more than seven times higher in the case of scraper saw blade, reaching *F*_*f*_*crit*_ = 3640 N.

Computer simulations revealed that the effect of the feed force *F*_*f*_ on changes in the operating stiffness *k*_*0w*_ is linear. It was also found that it is possible to determine values of the operating stiffness when the critical feed force *F*_*f*_*crit*_ is known. It follows the solution proposed by Timoshenko^52^ and can be expressed as in Eq. (6):

6 $$ k_{0w} = k_{0} \left( {1 - \frac{{F_{f} }}{{F_{f\_crit} }}} \right) $$

**Figure 9:**
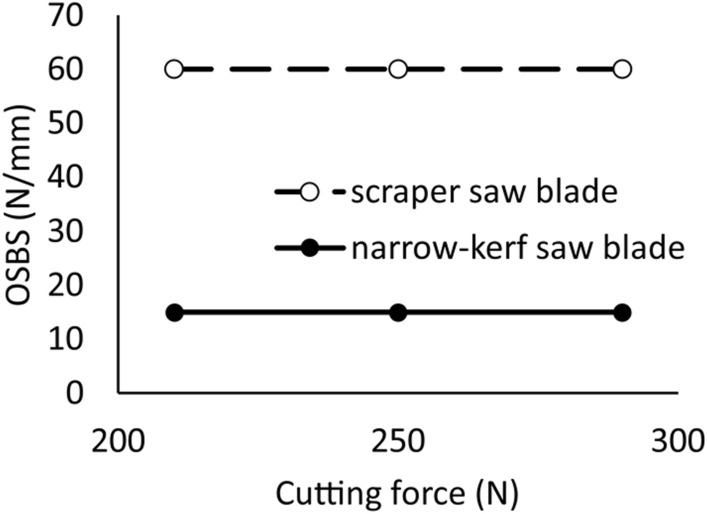
The operational saw blade stiffness OSBS of the narrow-kerf and scraper saw blades as a function of the cutting force (calculations for *F*_*p*_ = 100 N and *F*_*f*_ = 125 N).

Additional analyses were carried out to determine the effect of the cutting force *F*_*c*_ on the stiffness value of both investigated saw blade types. The results of *OSBS* numerical simulations are shown in Fig. 9 evidencing that the cutting force *F*_*c*_ does not affect the stiffness value of the saw blade. It confirms previous analyses carried out by Orlowski^68^ and Prokofiev^69^.

### Analysis of the (extreme) scraper saw blades behaviour during sawing of lamellae

**Figure 10:**
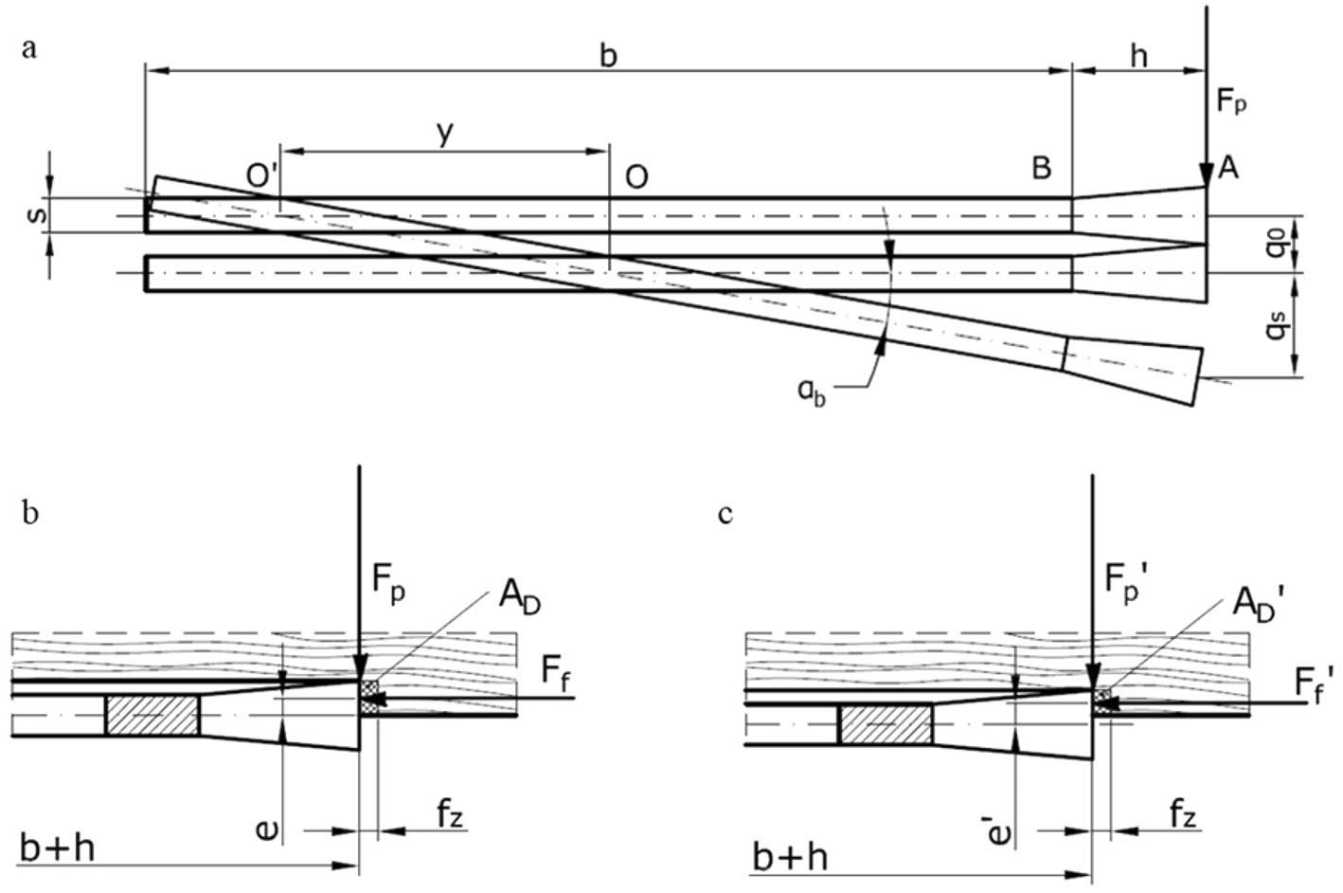
The transverse displacement of the saw blade under the action of the thrust force *F*_*p*_, where: *q*_0_ is the displacement resulting from the deflection of the saw blade corresponding to the displacement under the action of the *F*_*p*_ force applied at point *O*; *q*_*s*_ is the torsional displacement of the blade; *h* is the tooth height; *b* is the blade width; *s* is the blade thickness; *y* is the displacement of the pivotal point relative to the middle of blade *O*; *α*_*b*_ is the saw blade torsion angle; *A*_*D*_ and *A*_*D*_*’* is the cross-sections of the layers being cut.

Primary components of the tip total displacement for the saw blade tooth caused by the action of the thrust force *F*_*p*_ are presented in Fig. 10.

The actual pivotal point of the saw blade is located outside point *O.* It is shifted to *O’*, which is separated from point *O* by the distance *y*. The value of *y* can be determined from the relationship^47^ expressed in Eq. (7):

7 $$ y = \frac{{q_{0} }}{{q_{s} }}\left( {\frac{b}{2} + h} \right) $$

An understanding of the *y* value is indispensable for proper identification of torques and deflections, especially when cutting with loads applied asymmetrically to the saw blade teeth. It is a typical cutting configuration of the scraper saw that works with the material present only on one side of the blade. It can be assumed following the work^40^ that the ratio *q*_*0*_/*q*_*s*_ = 2/5 when considering narrow-kerf saw blades with a width (*b* + *h*) < 50 mm.

The saw blade tooth under analysis is loaded during cutting (in addition to the thrust force *F*_*p*_) with the feed force *F*_*f*_ applied to the saw blade at a distance *e* from point *A* that corresponds to *e* = *S*_*t*_/4 (Fig. 10b). This tooth is in contact with the material along the main cutting edge, with the contact length equal to the width of the layer being cut *S*_*t*_/2. The torque from the feed force *F*_*f*_ partially compensates an effect of the thrust force *F*_*p*_ action. It was noticed that the shift of the pivotal point was *y* = 9 mm in the case of the studied saw blade with *b* = 35 mm and *h* = 5 mm. The balance equation for the moments of the force relative to point *O’* is therefore dependent on the feed force *F*_*f*_ = 212 N, as shown in Eq. 8:

8 $$ F_{p} \cdot 31.5 = 212 \cdot \frac{2.9}{4} $$

The maximal thrust force that will not cause any saw blade displacement can be determined using Eq. (8) and corresponds to *F*_*p*_ = 4.88 N. Therefore, the theoretical track of the saw blade will deform when the thrust force increases over that value. It will decrease the dimensional accuracy for the lamellae being cut.

It was demonstrated^37^ that oak wood lamellae obtained from the outer positions in the frame sawing machine gang are thicker than the medial lamellae, despite the fact that the outer saws are thicker and thus stiffer. This is interpreted as a result of the asymmetric loads occurring on the scraper blades during the process of cutting the wood. An increase in the thickness of the outer lamella (by 0.2 mm for example) can be associated with the presence of natural heterogeneity in the raw material being sawn. The coefficient of working stiffness can be estimated here as in Eq. (9), following Timoshenko’s^52^ theorem (Eq. 6):

9 $$ k_{0w} = k_{0} \left( {1 - \frac{{F_{f} }}{{{3639}{\text{.8}}}}} \right) $$

Consequently, the working stiffness coefficient is *k*_0w_ = 57.24 N⋅mm^−1^, assuming the initial stiffness coefficient *k*_0_ = 60.78 N⋅mm^−1^ and the feed force *F*_*f*_ = 212 N. The value of the thrust force that will trigger a displacement of 0.2 mm is *F*_*p*_ = 0.2 *k*_0w_ = 11.45 N. It should be noticed, however, that the contact of the main cutting edge with the workpiece is reduced as a consequence of the saw displacement by 0.2 mm by the same value. It simultaneously results in a proportional decrease of the feed force *F*_*f*_. The width of the chip being cut drops to dimension *S*_*t*_/2 – 0.2 = 1.25 mm, resulting in the feed force *F*_*f*_ = 182.76 N. The point of application of such altered feed force will also change and become *e*_1_ = 0.825 mm (Fig. 10c). The load torques *M(F*_*p*_*)* and *M(F*_*f*_*)* can be determined for this position of the feed force following Eqs. (10) and (11), respectively:

10 $$ M\left( {F_{p} } \right) = { 11}.{45} \cdot {31}.{5 } = { 36}0.{\text{68 N mm}} $$

11 $$ M\left( {F_{f} } \right) = F_{f} \cdot e_{1} = { 182}.{76 } \cdot 0.{83 } = { 151}.{\text{69 N mm}} $$

The moment from the thrust force *M*(*F*_*p*_) is more than two times higher than that caused by the feed force *M*(*F*_*f*_). Moreover, there are no additional external forces within the cutting system which could return the scraper saw blade to its starting position. It is assumed, therefore, that only variations within the structure of the wood being sawn can trigger this phenomenon.

The analysis of the scraper saw blade behaviour and computation of its stiffness coefficients made it possible to explain the processing inaccuracies as observed when re-sawing oak lamellae^37^. Furthermore, it explains why high values of the feed speed are not used in industrial practice, especially when manufacturing wooden elements requiring high dimensional accuracy. Even though majority of sawing machines are capable of high process velocities, an increase of the feed speed increases the variation in thickness that may exceed accepted tolerances. It is especially noticed for the outer lamellae in the saw set, resulting in unnecessary material loss and a rise in production costs. In the latter case, these excess layers must be removed in the subsequent process steps, such as circumferential planning and/or sanding.

## Conclusions

The following conclusions can be drawn based on the results of the theoretical and finite element method analysis of the stiffness of the saw blades:

• The initial static stiffness of the saw blade has a linear course and is a function of the geometric dimensions of the saw blade and its preload or tensioning level.

• The scraper saws have an initial static stiffness that is three times higher than narrow-kerf saw blades when installed in the same gang.

• The feed force has a large effect on the stability of the saw blade and reduces the initial static stiffness of the saw blade to the operating stiffness. The loss of stability of the scraper saw blade can occur at the feed-per-tooth force exceeding *F*_*f*_*crit*_ = 3640 N. The corresponding critical force for the narrow-kerf saw blade is *F*_*f*_*crit*_ = 493 N.

• The calculation of the operating stiffness of saws as a function of the feed force can be performed using Timoshenko’s equations.

• The cutting force does not affect the value of the operating stiffness of the saw.

• The effect of asymmetric loads when sawing wood on scraper saw blades can cause severe sawing inaccuracies, particularly in the outer lamellae in the gang.

The simulation methodology developed in this research can be straightforwardly extended towards the determination of optimal geometry of other cutting tools, particularly focusing on kerf reduction. It may lead to more sustainable use of natural resources as well as an increase in economic gain for the wood processing industries.
